# Comparative analysis of the efficacy of vaccines using structural protein subunits of the severe fever with thrombocytopenia syndrome virus

**DOI:** 10.3389/fmicb.2024.1348276

**Published:** 2024-03-19

**Authors:** Sohee Kim, Kyeongseok Jeon, Hooncheol Choi, Da-Eun Jeong, Jun-Gu Kang, Nam-Hyuk Cho

**Affiliations:** ^1^Department of Microbiology and Immunology, College of Medicine, Seoul National University, Seoul, Republic of Korea; ^2^Department of Biomedical Sciences, College of Medicine, Seoul National University, Seoul, Republic of Korea; ^3^Korea Zoonosis Research Institute, Jeonbuk National University, Iksan, Republic of Korea; ^4^Institute of Endemic Disease, Seoul National University Medical Research Center, Seoul, Republic of Korea; ^5^Seoul National University Bundang Hospital, Seongnam, Republic of Korea

**Keywords:** severe fever with thrombocytopenia syndrome (SFTS), SFTS virus (SFTSV), vaccine, subunits, memory

## Abstract

The severe fever with thrombocytopenia syndrome virus (SFTSV) represents a significant emerging health threat as a tick-borne pathogen that causes SFTS, with mortality rates ranging between 10 and 30%. Despite the considerable risk presented by SFTSV, an effective vaccine has yet to be developed. Our study assessed the efficacy of recombinant protein vaccines, focusing on the purified nucleocapsid protein (NP) and surface glycoproteins (Gn and Gc), against SFTSV in both singular and combined formulations. Individual vaccinations with NP or Gn subunits yielded partial protection in type I interferon receptor-knockout (IFNAR-KO) mice, with survival rates of 66.7 and 16.7%, respectively, whereas Gc vaccination did not confer significant protection, resulting in 100% mortality similar to that of the unvaccinated control group. Notably, NP vaccination substantially enhanced antigen-specific T cell responses, and Gc vaccination exhibited strong neutralizing activity against SFTSV. Among the combined recombinant protein formulations (Gn + NP, Gc + NP, and Gn + Gc + NP) tested, the Gc + NP combination provided the highest survival rate (85.7%) following challenge with a lethal dose of SFTSV, highlighting its potential as a vaccine candidate. Longitudinal studies showed that antibody levels in both wild type C57BL/6 and IFNAR-KO mice peaked between 2 and 3 months post-vaccination and declined over time. A notable decrease in NP-specific CD8^+^ T cell responses was observed 6 months post-vaccination in C57BL/6 mice, while NP-specific CD4^+^ T cell responses persisted up to 12 months. By 12 months post-vaccination, all IFNAR-KO mice vaccinated with single subunit antigens succumbed to the virus, suggesting that effective protection against SFTS may rely on antibody responses to subunit antigens and/or CD8^+^ T cell activity. These findings underscore the necessity of an optimized SFTS vaccine that combines protective antigens with an adjuvant system to ensure durable humoral and cellular immunity.

## 1 Introduction

Severe fever with thrombocytopenia syndrome (SFTS) is a rising tick-borne zoonotic illness caused by the SFTS virus (SFTSV, also known as *Dabie bandavirus*), belonging to the Bunyavirales order, and first identified in a patient in China (Yu et al., 2011). SFTSV’s genome consists of three segments of negative or ambisense single-stranded RNA: the S segment, encoding a nucleoprotein (NP) and a nonstructural protein (NS); the M segment, encoding a glycoprotein precursor (split into glycoproteins Gn and Gc); and the L segment, encoding an RNA-dependent RNA polymerase (Yu et al., 2011). The primary vector for SFTSV, *Haemaphysalis longicornis*, is prevalent in Eastern Asia, with tick bites being a major route of transmission (Reece et al., 2018). However, human and animal infections can also arise through direct contact with infectious blood or bodily fluids (Tang et al., 2013; Jung et al., 2019; Fang et al., 2021). SFTS symptoms include high fever, thrombocytopenia, leukocytopenia, and in severe cases, multiorgan dysfunction. Reported case fatality rates in China, Japan, and South Korea are ranging from 10 to 30%, depending on patients’ age, sex, and endemic regions (He et al., 2020; Miao et al., 2021; Jeon et al., 2023), with the virus’s presence and its antibodies also noted in various Southeast Asian countries, raising concerns about its spread (Tran et al., 2019; Peng et al., 2020; Win et al., 2020; Rattanakomol et al., 2022).

To understand SFTSV pathogenicity and develop antiviral drugs or vaccines, experimental animal models are vital (Sun et al., 2021). Although SFTSV can infect various species (Oh et al., 2016; Kang et al., 2019; Matsuu et al., 2019), most immunocompetent experimental animals do not show severe disease (Sun et al., 2021). Researchers have developed SFTSV-susceptible animal models using type 1 interferon (IFN) receptor-knockout (IFNAR-KO) mice, STAT2-deficient golden Syrian hamsters, and aged ferrets and cats, with IFNAR-KO mice and aged ferrets being primary models for vaccine development (Liu et al., 2014; Gowen et al., 2017; Matsuno et al., 2017; Yu et al., 2019; Kang et al., 2020). Recombinant vesicular stomatitis virus-based, attenuated vaccinia virus-based, and plasmid DNA vaccines have shown complete protection in these models (Dong et al., 2019; Kwak et al., 2019; Yu et al., 2019; Kang et al., 2020; Yoshikawa et al., 2021).

Nucleoproteins (NPs) are highly immunogenic, and NP-specific antibodies are detectable post-infection, making NP-based ELISA or S-segment targeted PCR common diagnostic tools for SFTSV (Li et al., 2018; Thi Hai Yen et al., 2019). However, despite their immunogenicity, NPs of several bunyaviruses do not elicit virus-neutralizing antibodies (Boshra et al., 2011). Our previous study has also shown that IFNAR-KO mice vaccinated with SFTSV NP demonstrated partial survival after challenge, despite lacking virus-neutralizing activity (Kang et al., 2020). In contrast, mice immunized with the Gc protein developed high titers of virus-neutralizing antibodies but did not achieve protection against SFTSV. Therefore, the correlation between the viral proteins and cell-mediated or humoral responses, as well as their impact on the clinical outcomes of SFTSV, remains unclear. In the present study, we explore the development of recombinant protein vaccine candidates, including purified NP and Gc and Gn proteins against SFTSV demonstrating their short-to long-term immunogenicity and protection against SFTSV infection in IFNAR-KO mice.

## 2 Materials and methods

### 2.1 Ethics statement

All animal experiments were carried out in the Animal Biosafety Level 3 facility of Seoul National University Hospital and the Korea Zoonosis Research Institute. The studies received approval from the Institutional Animal Care and Use Committees (IACUC) of Seoul National University Hospital and Jeonbuk National University (SNUH IACUC No. 21-0288-S1A1 and JBNU IACUC No. 2020-0142), and were conducted in strict adherence to the recommended guidelines for the care and use of laboratory animals.

### 2.2 Preparation of recombinant proteins

The genes encoding Gn and Gc from the KASJH strain (GenBank Accession No. KP663732, Supplementary Table S1) were cloned into a pcDNA3 vector (Addgene, Watertown, MA, United States). These vectors were transfected into HEK293F cells (Thermo Fisher Scientific, Waltham, MA, United States) using polyethylenimine. Following transfection, the cells were cultured in FreeStyle 293 expression medium (Gibco, Waltham, MA, United States) for 5 days. Recombinant proteins collected from the supernatants were purified using HisTrap HP histidine-tagged protein columns (GE Healthcare, Chicago, IL, United States) with the AKTA start^™^ system (GE Healthcare), following the manufacturer’s instructions. The SFTSV NP recombinant protein purification process was conducted as previously outlined (Kang et al., 2020). Briefly, the pET28a(+) vector cloned with the gene encoding SFTSV NP was transfected into the *Escherichia coli* strain BL21 (DE3). The NP protein, overexpressed after 18 h of induction with isopropyl β-D-thiogalactoside at 16°C, was purified using HisTrap HP histidine-tagged protein columns (GE Healthcare) as per the manufacturer’s guidelines. All proteins were confirmed to contain less than 0.05 EU/mg of endotoxin. The identity and purity of the proteins, verified to be greater than 90%, were determined through western blotting and coomassie blue staining, respectively (Supplementary Figure S1). The purity of the purified antigens was assessed by measuring their optical intensity relative to the background intensity, utilizing ImageJ software (Schneider et al., 2012).

### 2.3 Enzyme-linked immunosorbent assays

To assess the antibody titers against recombinant SFTSV proteins in the sera of immunized and non-immunized (mock) mice, immunoassay plates (96-well plates; Nunc, Rochester, NY, United) were prepared by coating each well with 100 ng of purified antigens and incubating overnight at 4°C. Following the antigen coating, the plates were washed with PBS containing 0.05% Tween 20 (PBST; Sigma-Aldrich, St. Louis, MO, United States) and subsequently blocked for 2 h at room temperature using PBST mixed with 5% skim milk (BD Difco, Sparks, MD, United States). Serum samples from each mouse were serially diluted in a 4-fold increment across 8–10 points starting at a 1:100 dilution, with 100 μL of these diluted samples then incubated for 1 h at room temperature in the plates. Sera from pre-immunized mice served as negative controls to establish the cut-off titer (mean O.D. ± 3 × S.D. at 1:100 diluents). For detection, horseradish peroxidase-conjugated goat anti-mouse IgG antibodies (diluted 1:10,000; Thermo Fisher Scientific) were employed as secondary antibodies. Plates were then washed with 0.05% PBST before adding 3,3′,5,5′-tetramethylbenzidine peroxidase substrate solution (BioLegend, San Diego, CA, United States) to develop the color for 7 min. The reaction was stopped with a 1 M H_3_PO_4_ solution, and the absorbance was measured at 450 nm using a microplate reader (TECAN, Mannedorf, Switzerland).

### 2.4 Flow cytometric analysis

Spleens collected from mice were processed by grinding and filtering through a 70 μm cell strainer (BD Biosciences, San Jose, CA, United States), and the resulting splenocytes were suspended in RPMI 1640 media (Gibco) supplemented with 10% FBS (Gibco) and 1% penicillin/streptomycin (Gibco). Following lysis of red blood cells with Red Blood Cell lysing buffer Hybri-Max^™^ (Sigma-Aldrich), the splenocytes were cultured in complete RPMI medium (enhanced with 10% FBS and 1% penicillin/streptomycin solution) at a density of 10 μg of purified NP, Gn, and Gc proteins per well for 18 h at 37°C with 5% CO_2_ on a 96-well cell culture plate, with each experiment duplicated for individual mice. For intracellular IFN-γ detection, splenocytes (2 × 10^6^ cells/well) were treated with 1 μg of Golgi-plug (BD Bioscience) for the last 5 h of incubation. Cells were first stained with Zombie aqua fixable dye (BioLegend) and then blocked with ultra-block solution containing 10% rat sera, 10% hamster sera, and 10% mouse sera (Sigma-Aldrich). Following blocking, cells were incubated with fluorescent dye-conjugated antibodies against CD3 (145-2c11; BD Biosciences), CD4 (RM4-5; BD Biosciences), and CD8 (53-6.7; BioLegend) for 30 min at room temperature. After surface staining, cells were fixed and permeabilized using the Cytofix/Cytoperm kit (BD Biosciences), allowing for the intracellular staining with anti-IFN-γ antibody (XMG1.2; BD Pharmingen, Franklin Lakes, NJ, United States). The stained cells were analyzed using a CytoFLEX S flow cytometer (Beckman Coulter, Brea, CA, United States), and the flow cytometry data were processed using FlowJo software version 10.8 (Tree Star, Ashland, OR, United States). The gating strategy is presented in Supplementary Figure S2.

### 2.5 Preparation of SFTSV

The SFTSV strain 2015-JJ01 (NCBI Genbank accession numbers MN329148-MN329150) was cultured in VeroE6 cells (ATCC No. CRL-1586), as described previously (Kang et al., 2020). The focus-forming units (FFU) of SFTSV were quantified using a focus forming assay with methylcellulose media. In brief, viral supernatants were filtered, serially diluted in 10-fold increments, and then applied to a monolayer of VeroE6 cells. After incubation for 1 h at 37°C, the supernatants were discarded, and the cells were overlaid with a medium consisting of DMEM supplemented with 2% FBS and 1% methylcellulose, followed by incubation at 37°C for 6 days. Subsequently, the cells were fixed and permeabilized using 100% methanol for 20 min at room temperature. SFTSV foci were identified with a rabbit anti-SFTS NP antibody (AbClon, Seoul, Republic of Korea) and a goat anti-rabbit IgG secondary antibody conjugated to horseradish peroxidase (Invitrogen, Waltham, MA, United States). The presence of viral foci was visualized through incubation with 3,3′-diaminobenzidine (DAB) Substrate (Merck, Darmstadt, Germany).

### 2.6 Virus neutralizing antibody assay

To assess the neutralizing efficacy of sera from immunized mice, a 50% focus reduction neutralization titer (FRNT50) assay was utilized. Sera were first inactivated by heating at 56°C for 30 min and then diluted four-fold across four points, ranging from 1:40 to 1:2560. Each serum dilution was mixed and incubated with an equal volume of SFTSV solution (containing 100 FFU) at 4°C for 1 h. This inoculated mixture was then applied to a monolayer of VeroE6 cells housed in a 24-well plate (SPL Life Sciences, Pocheon, Republic of Korea) and incubated for 2 h at 37°C. After this period, supernatants were discarded, and cells were cultured in an overlay medium (DMEM supplemented with 2% FBS, 1% penicillin/streptomycin, and 1% methylcellulose) at 37°C for 6 days. The process for visualizing viral foci is as described above. Percentage focus reduction was calculated as [(No. of plaques without antibodies) − (No. of plaques with antibodies)]/(No. of plaques without antibodies) × 100. All the experiments were duplicated and the FRNT_50_ was calculated using nonlinear regression analysis (log[inhibitor] versus normalized response method) embedded in GraphPad Prism Software v8.0 (GraphPad Software; https://www.graphpad.com). All FRNT50 assays were conducted alongside negative control sera to establish the limit of detection under our experimental conditions.

### 2.7 Immunization of mice and the SFTSV challenge

Wild type C57BL/6 female mice aged between 6 to 8 weeks (Orient Bio Inc., Seongnam, Republic of Korea), as well as IFN α/β receptor-knockout (IFNAR-KO) mice on a C57BL/6 background (Min et al., 2018), were utilized for immunization and subsequent challenge experiments. These mice were kept in a specific pathogen-free environment at either the Seoul National University College of Medicine or the Korea Zoonosis Research Institute of Jeonbuk National University. For immunization, mice received subcutaneous injections of 10 μg of recombinant SFTSV NP-His, Gn-His, and Gc-His proteins, combined with aluminum hydroxy chloride (alum; Alhydrogel^®^ adjuvant 2%; InvivoGen, Hong Kong), administered three times at two-week intervals. Serum samples were collected from the mice weekly following each immunization and then every months for a year after the last immunization. Two weeks after receiving the final dose of vaccine, the mice were exposed to a challenge dose of 10^3^ FFU of SFTSV (strain 2015-JJ01). Blood samples were taken 4 days post-infection (dpi), and both survival rates and body weight changes were monitored and recorded up to 14 dpi.

### 2.8 Quantitative reverse transcript-polymerase chain reaction

Total RNA was isolated from the blood of SFTSV-infected mice utilizing TRIzol LS reagent (Life Technologies, Carlsbad, CA, United States), according to the manufacturer’s guidelines. Blood samples underwent lysis employing a TissueLyser II (Qiagen, Hilden, Germany), operating at a frequency of 30 Hz for a duration of 5 min, to facilitate the RNA extraction process. Subsequently, the harvested RNA was converted into cDNA using the HiSenScript RH (−) RT Premix kit (Intron, Seongnam, Republic of Korea). Quantification of the resultant cDNA was achieved through the application of TaqMan Universal Master Mix II (Applied Biosystems, Waltham, MA, United States). Quantitative reverse transcript-polymerase chain reaction (qRT-PCR) analysis was conducted using a BioRad CFX connect real-time PCR system (Bio-Rad, Hercules, CA, United States), adhering to the following thermal cycling conditions: an initial uracil-N-glycosylase (UNG) incubation step at 50°C for 2 min, followed by polymerase activation at 95°C for 10 min, denaturation at 95°C for 15 s, and then annealing/extension at 53°C for 1 min, across a total of 40 cycles. Fluorogenic signals were captured during the annealing/extension phase. The specific primer set and probe utilized for detecting the NP gene of SFTSV in the qRT-PCR were as follows: NP forward primer (5′-CCTTCAG GTCATGACAGCTGG-3′), NP reverse primer (5′-ACCAGGCTCTCAATCACTCCTGT-3′), and the fluorogenic detecting probe (5′-6FAM-AGCACATGTCCAAGTGGGAAGGCTCTG-BHQ1-3′). The quantification of viral RNA copy numbers was determined relative to a standard control, enabling precise measurement of the viral load in the samples.

### 2.9 Hematology

For the purpose of hematological analysis, animals were euthanized and subjected to blood collection via the eye-bleeding technique. The collected blood samples were immediately placed in tubes pre-coated with 0.5 M ethylenediaminetetraacetic acid (EDTA) (Enzynomics, Daejeon, Republic of Korea) to prevent coagulation. To ensure the inactivation of any viruses present, a solution of 4% paraformaldehyde was added to the EDTA-anticoagulated whole blood samples at a 1:1 volume ratio prior to the assessment of platelet counts. The analysis of platelet counts was conducted using the BC-2800 Vet Auto Hematology Analyzer (Mindray Bio-Medical Electronics Co., Ltd., China).

### 2.10 Statistical analysis

Data analysis was conducted utilizing GraphPad Prism version 8.0 (GraphPad Software). For statistical evaluations, a two-tailed Mann–Whitney *U* test or a Kruskal–Wallis test followed by Dunn’s multiple comparisons test was employed to assess differences among various groups. The results are presented as the mean ± standard deviation (S.D.). The analysis of survival rates was carried out using the Mantel–Cox log rank test. A *p*-value of less than 0.05 was deemed to indicate statistical significance.

## 3 Results

### 3.1 Humoral and cell-mediated immune responses to viral antigens in wild type and IFNAR-KO mice immunized with single recombinant viral protein subunit

**Figure 1:**
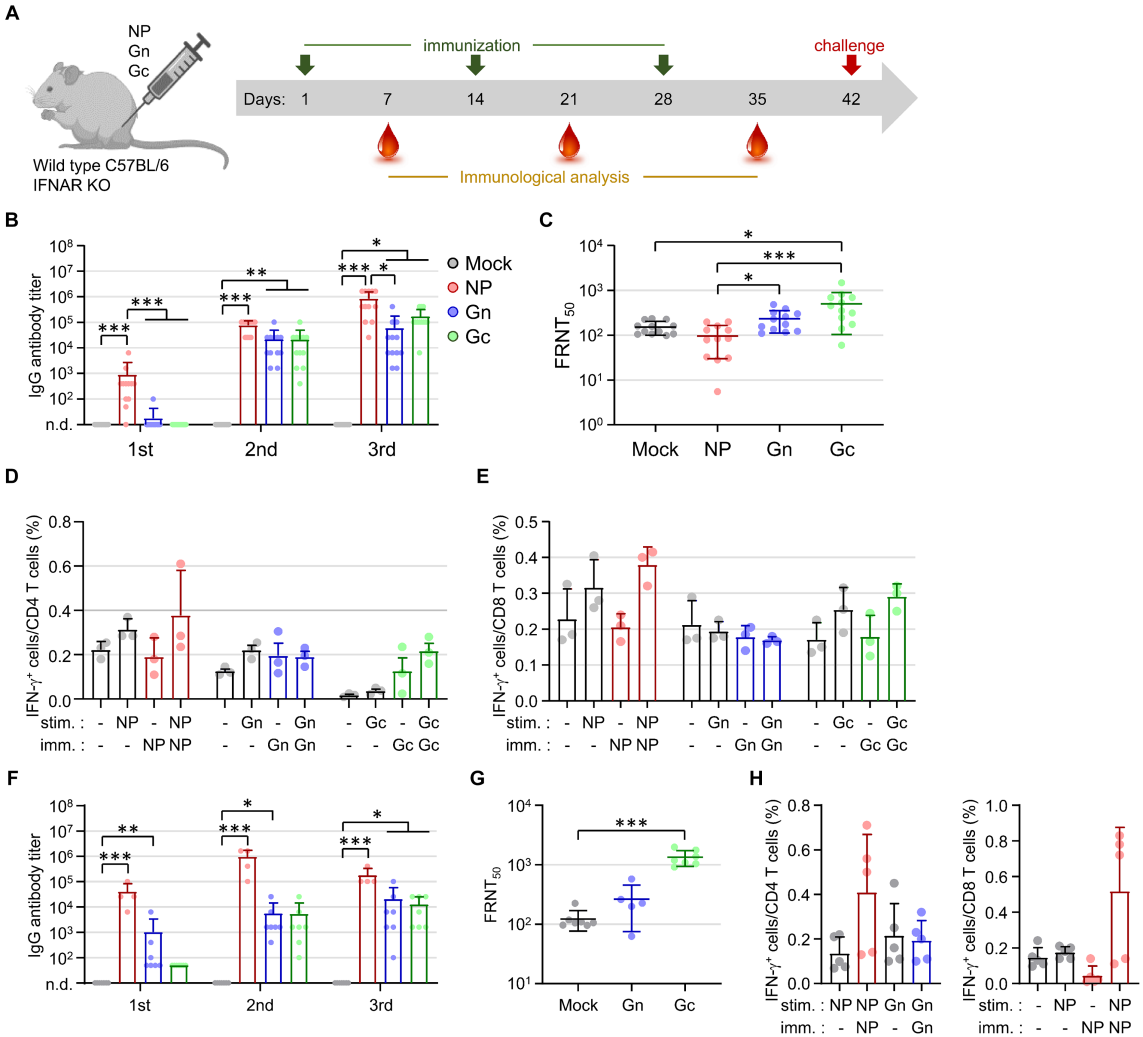
Assessment of antigen-specific antibody and T cell responses in IFNAR-KO and wild type C57BL/6 mice following immunization with recombinant SFTSV proteins. **(A)** Timeline detailing the immunization schedule, blood collection intervals, and SFTSV challenge protocol. IFNAR-KO or wild Type C57BL/6 mice were administered alum alone or alum combined with one of the recombinant proteins (NP, Gn, or Gc). **(B)** The IgG responses to NP, Gn, or Gc were quantified using ELISA 1 week post each immunization dose. The graph displays the antibody titers for each recombinant protein (NP, Gn, or Gc) in the sera from IFNAR-KO mice (*n* = 12/group) immunized with the respective protein plus alum or with alum alone (mock). No specific antibody response was detected in the sera from mock-immunized mice. **(C)** The presence of serum neutralizing antibodies against SFTSV in IFNAR-KO mice was determined 1 week after the final immunization, employing the FRNT_50_ method. **(D,E)** At 1 week post the last immunization, splenocytes were isolated from IFNAR-KO mice (*n* = 3/group) immunized with each recombinant protein. The production of IFN-γ by CD4^+^ T cells **(D)** and CD8^+^ T cells **(E)** was evaluated through flow cytometry following stimulation with the specified antigens. **(F)** The IgG response to NP, Gn, or Gc in wild type C57BL/6 mice (*n* = 4 - 7/group), immunized with the respective protein plus alum or with alum alone (mock), was measured using ELISA 1 week after each immunization. Specific antibody titers were reported for each group. No specific antibody response was detected in the sera from mock-immunized mice. **(G)** Serum neutralizing antibodies targeting SFTSV in C57BL/6 mice were assessed 1 week following the final immunization using the FRNT_50_ criterion. **(H)** Splenocytes harvested from C57BL/6 mice (*n* = 5/group) immunized with each recombinant protein 1 week after the final immunization were analyzed for NP-specific, IFN-γ-producing CD4^+^ T cells (left) or CD8^+^ T cells (right) via flow cytometry. Error bar, mean ± S.D.; ^*^p < 0.05, ^**^p < 0.01, and ^***^p < 0.001.

To evaluate the immune responses elicited by viral antigens, wild type C57BL/6 mice and IFNAR-KO mice were immunized subcutaneously with 10 μg of recombinant NP, Gn, or Gc proteins, each formulated with alum, at two-week intervals for a total of three doses (Figure 1A). Following immunization, IFNAR-KO mice exhibited a significant increase in antibody responses to the NP antigen after each of the three doses (Figure 1B). In contrast, antibody responses to Gn or Gc antigens in IFNAR-KO mice immunized with these proteins did not show an increase after the first dose but demonstrated significant elevation after the second and third doses (Figure 1B). Neutralizing antibody levels against SFTSV were measured in sera 1 week post-final immunization. The neutralizing antibody titers (FRNT50) in IFNAR-KO mice immunized with Gn were modestly higher than those in the control group (mean = 232.8 vs. 151.9), and titers were significantly higher in the Gc-immunized group (mean = 498.9) compared to the controls (Figure 1C). However, neutralizing antibody titers in mice immunized with NP (mean = 96.9) were comparable to those in the control group. These findings indicate that while the NP antigen is highly immunogenic for inducing an antibody response, it is barely effective at inducing neutralizing activity against SFTSV.

The analysis of antigen-specific T cell responses in IFNAR-KO mice immunized with individual recombinant proteins was conducted by evaluating IFN-γ secretion. Splenocytes from immunized mice were stimulated with the corresponding immunizing antigens (NP, Gn, or Gc) 1 week after the final immunization. The frequency of IFN-γ-producing CD4^+^ or CD8^+^ T cells in response to NP or Gc antigens showed a slight increase in the groups of mice immunized with the corresponding antigens compared to the mock-immunized mice; however, these increases were not statistically significant (Figures 1D,E). In addition, the frequencies of IFN-γ-producing CD4^+^ or CD8^+^ T cells in response to Gn did not show significant differences between the immunized mice and control group. This suggests that the vaccine formulation, comprising a viral subunit protein and alum hydroxide adjuvant, may not effectively generate cellular immunity against the viral antigens.

Investigating the potential cause of the weak cellular immunity in IFNAR-KO mice, we conducted parallel experiments in wild type C57BL/6 mice (Figures 1F–H). The patterns of humoral and cellular immune responses observed in the wild type mice, following immunization with the subunit antigens, were similar to those seen in IFNAR-KO mice. This confirmed the superior immunogenicity of the NP antigen for antibody responses and the significant induction of neutralizing activity by Gc immunization. The frequencies of NP-specific CD4^+^ or CD8^+^ T cell responses were substantially higher in NP-immunized mice compared to those in mock groups, yet these increases did not achieve statistical significance (Figure 1H). This consistently underscores the subunit antigen’s limited capacity to elicit cellular immunity, even in wild-type mice, when paired with the alum hydroxide adjuvant.

### 3.2 Partial protection against SFTSV infection in IFNAR-KO mice through vaccination with single subunit recombinant proteins

**Figure 2:**
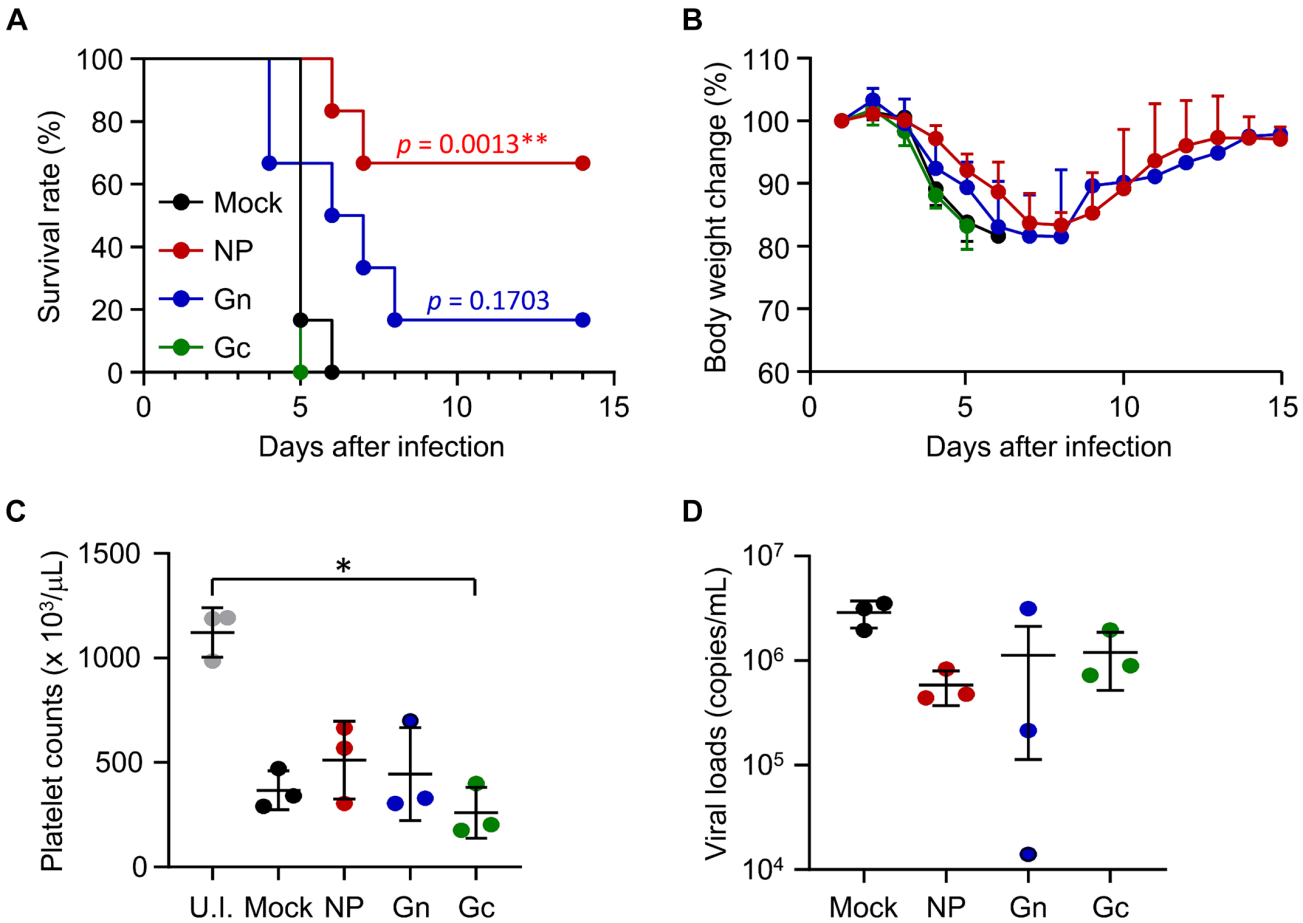
Protective efficacy of recombinant SFTSV proteins in IFNAR-KO mice against lethal SFTSV challenge. **(A)** Survival rates and **(B)** body weight changes are shown for mice (*n* = 6/group) that were immunized three times with specified recombinant proteins and then challenged with 10^3^ FFU of SFTSV 2 weeks after the final immunization. The statistical significance of enhanced survival due to vaccination was assessed through pair-wise comparison with the mock group, using the Mantel–Cox log rank test. **(C)** Platelet counts and **(D)** viral loads in the blood were assessed for mice (*n* = 3/group) immunized with the indicated recombinant proteins. Blood samples were collected 4 days post-infection. U.I., uninfected. Error bar, mean ± S.D.; ^*^p < 0.05 and ^**^p < 0.01.

To assess the protective efficacy of vaccination with recombinant protein subunits, IFNAR-KO mice immunized with NP, Gn, or Gc proteins formulated with alum hydroxide received subcutaneous challenges with 10^3^ FFU of SFTSV. Following infection, all mice in the mock and Gc-immunized groups succumbed by days 6 and 5, respectively (Figure 2A). In contrast, four out of six mice (66.7%) vaccinated with NP and one out of six mice (16.7%) immunized with Gn survived the challenge. These surviving mice experienced a gradual loss of body weight until 6dpi before regaining their weight thereafter (Figure 2B). At 4 dpi, a substantial reduction in platelet counts was observed across all infected mice, with Gc-immunized mice showing a notably lower platelet count compared to uninfected control mice (Figure 2C). Although viral loads in blood of immunized mice generally decreased by 4 dpi, these reductions were not statistically significant when compared to mock-immunized controls (Figure 2D). Consequently, the strong antibody response and moderate T cell reactivity elicited against the NP antigen by the subunit vaccine may contribute to significant improvement in the survival rate of mice lethally challenged with SFTSV. However, such protective effects were not achieved through immunization with either the Gn or Gc antigens in our vaccine formulation.

### 3.3 Enhanced protection against SFTSV infection in IFNAR-KO mice through combined immunization with viral subunit proteins

**Figure 3:**
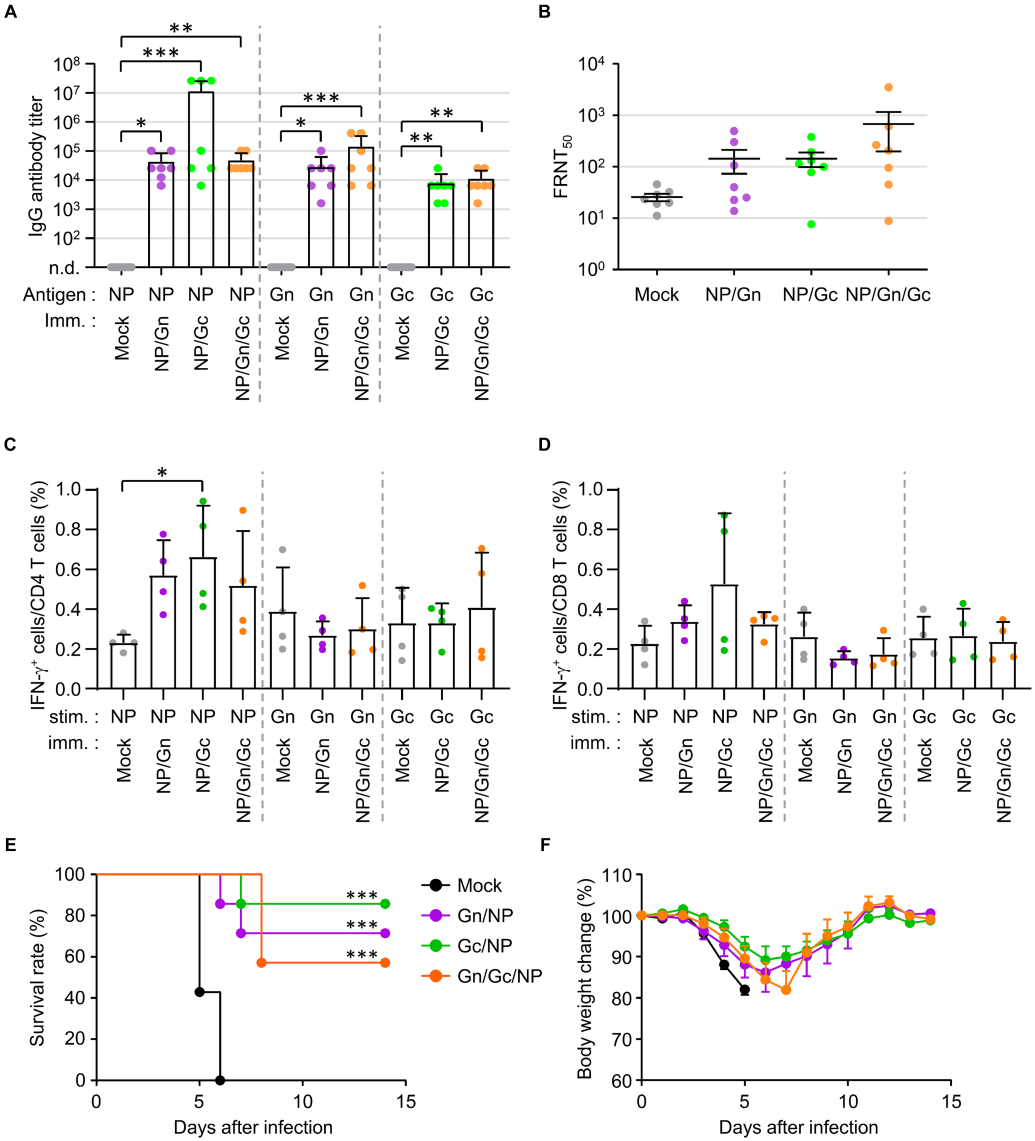
Antibody and T cell responses in IFNAR-KO mice immunized various combinations of SFTSV subunit antigens and their protection against lethal SFTSV challenge. **(A)** Antigen-specific IgG responses in each combinatorial vaccine group were measured using ELISA at 1 week after the third immunization. The antibody titers of anti-NP, Gn, or Gc IgG in sera of immunized mice (*n* = 7/group) are presented. Cut-off titers were determined using sera from pre-immunized mice. **(B)** Serum neutralizing antibodies against SFTSV in IFNAR-KO mice (*n* = 7/group) were evaluated based on FRNT_50_ assay. **(C,D)** Splenocytes were collected from IFNAR-KO mice (*n* = 4/group) immunized with each recombinant protein combination at 1 week after the third immunization. IFN-γ-secreting CD4^+^ **(C)** or CD8^+^ T **(D)** cells were analyzed using flow cytometry after simulation with the indicated antigens. **(E,F)** Survival rates **(E)** and body weight changes **(F)** were monitored in mice (*n* = 7/group) immunized with the specified combinations of recombinant proteins, followed by challenge with 10^3^ FFU of SFTSV at 2 weeks after the final immunization. The statistical significance of enhanced survival due to vaccination was assessed through pair-wise comparison with the mock group, using the Mantel–Cox log rank test. Error bar, mean ± S.D.; ^*^p < 0.05, ^**^p < 0.01, and ^***^p < 0.001.

To investigate the potential synergistic effects of combined vaccination with recombinant SFTSV proteins on the morbidity and mortality of mice challenged with a lethal SFTSV infection, we evaluated the humoral and cellular immune responses in IFNAR-KO mice immunized with various combinations of recombinant SFTSV proteins. Mice were vaccinated three times at two-week intervals with the specified mixtures of recombinant proteins, after which antibody and T-cell responses were analyzed in sera and spleens, respectively, 1 week post the third vaccination. Antibody responses against the specified antigens were significantly heightened in all immunized groups, with a notable increase in anti-NP IgG levels observed in some mice (three out of seven) immunized with NP/Gc, compared to the mock group (Figure 3A). However, no significant differences were found in NP, Gn, or Gc-specific antibodies among the different immunization groups. The FRNT50 values in sera from mice immunized with the NP/Gn/Gc combination showed the highest mean (678.4 ± 1268.3, *n* = 7) compared to the NP/Gn (143.7 ± 185.4), NP/Gc (143.5 ± 118.2), and mock (25.7 ± 11.1) groups, although there were individual variations within each group and the group differences were not statistically significant (Figure 3B). The frequency of IFN-γ-secreting CD4^+^ or CD8^+^ T cells in response to NP was substantially increased in all immunized groups compared to non-immunized controls (Figures 3C,D). Notably, the frequency of NP-specific IFN-γ-producing CD4^+^ cells in the NP/Gc group was significantly higher than in the mock group. However, the frequency of Gn-or Gc-specific IFN-γ-producing T cells in all immunized groups did not show a significant increase compared to non-immunized controls.

The protective efficacy of the combined recombinant proteins against lethal SFTSV infection was evaluated by monitoring survival rates and body weight changes (Figures 3E,F). Survival rates significantly improved in all immunized groups compared to non-immunized controls, where all mice succumbed to the virus by day 6 post-infection. The NP/Gc group exhibited the highest survival rate (85.7%), followed by the NP/Gn group (71.4%) and the NP/Gn/Gc group (57.1%). Furthermore, the NP/Gc group experienced relatively milder disease morbidity, as evidenced by less severe body weight loss, compared to other immunized and mock groups, although the differences were slight (Figure 3F).

### 3.4 Longevity of the adaptive immune response in mice immunized with viral subunit proteins

**Figure 4:**
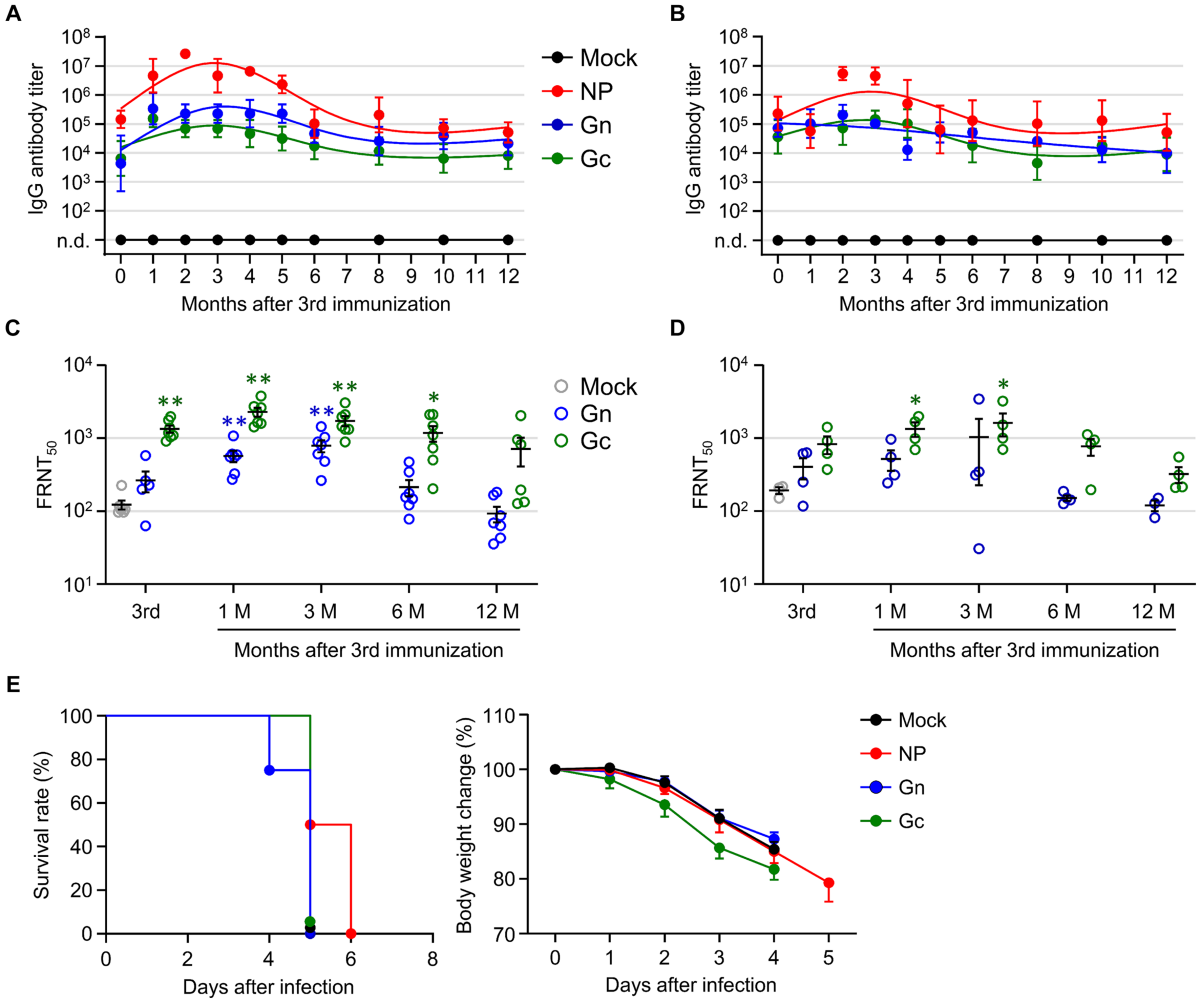
Evaluation of long-term immunity in mice immunized with recombinant SFTSV proteins. **(A,B)** The IgG responses to NP, Gn, and Gc were measured in wild type C57BL/6 **(A)** and IFNAR-KO **(B)** mice using ELISA on immune sera collected at specified intervals following the third immunization (*n* = 4–7/group). Cut-off titers were established based on sera from mice prior to immunization. **(C,D)** The presence of serum neutralizing antibodies against SFTSV in C57BL/6 **(C)** and IFNAR-KO mice **(D)** was determined using the FRNT_50_ assay for up to 1 year after the third immunization (*n* = 4–7/group). **(E)** Survival rates (left) and body weight changes (right) of IFNAR-KO mice (*n* = 6/group) immunized three times with each recombinant protein and challenged with 10^3^ FFU of SFTSV at 1 year after the final immunization. Error bar, mean ± S.D.; ^*^p < 0.05 and ^**^p < 0.01.

To investigate the longevity of the humoral immune response in mice immunized with the recombinant SFTSV proteins, we analyzed antibody titers against NP, Gn, and Gc antigens, as well as neutralizing antibodies against SFTSV in immune sera from wild type C57BL/6 and IFNAR-KO mice over a period of 1 year. To confirm whether C57BL/6 mice exhibited similar patterns of antigen-specific antibody titers and neutralizing titers, they were immunized with each single subunit recombinant protein (NP, Gn, or Gc) in the same manner. Both immunized C57BL/6 and IFNAR-KO mice displayed parallel trends in antibody titers, peaking 2–3 months after the final immunization and gradually declined (Figures 4A,B). Notably, in both mouse strains, the NP group showed a higher antigen-specific antibody titer than the Gn and Gc groups. The FRNT_50_ titers in sera from C57BL/6 mice immunized with Gn and Gc were significantly higher than those in the mock group, peaking at 1 to 3 months after the final immunization (Figure 4C). Similarly, IFNAR-KO mice groups showed a comparable pattern of neutralizing antibody levels, with the FRNT_50_ titers in Gc immunized IFNAR-KO mice being significantly higher than those in mock group at one to 3 months after immunization (Figure 4D). Across the observation period, groups vaccinated with Gc consistently showed higher neutralizing titers than those vaccinated with Gn. Nevertheless, this neutralizing activity began to decline 3 months post-immunization and became insignificant between 6 to 12 months after immunization in both immunized C57BL/6 and IFNAR-KO mice. When we assessed long-term T cell responses in NP-immunized C57BL/6 mice, the frequency of NP-specific IFN-γ-producing CD4^+^ T cells persistently elevated up to 12 months after immunization, but the levels were not significantly higher than those of non-immunized controls (Supplementary Figure S3). In case of IFN-γ-positive CD8^+^ T cells specific to NP antigen, they peaked at 3 months after the final immunization and sharply declined to baseline levels at 6 to 12 months.

Finally, we evaluated the enduring protective capacity of the recombinant protein vaccines in IFNAR-KO mice by exposing them to a lethal challenge dose of 1 × 10^3^ FFU of SFTSV at 12 months after their last immunization. Although the group vaccinated with NP exhibited a slightly prolonged survival compared to the other vaccinated groups and non-immunized controls, all the immunized mice ultimately succumbed to the viral challenge (Figure 4E), indicating that the subunit vaccines confer limited durability in terms of long-term immunity against SFTS.

## 4 Discussion

In our prior investigation, the administration of recombinant proteins from SFTSV glycoproteins Gn and Gc, fused with the Fc domain to IFNAR-KO mice, elicited significant humoral immune responses, including neutralizing activities (Kang et al., 2020). In the current study, we extended this observation to include His-tagged versions of the Gn and Gc antigens, administering them to both wild type C57BL/6 and IFNAR-KO mice. Our findings confirmed a marked enhancement in humoral immune responses (Figures 1, 4). Despite these promising results, vaccination with the Gc protein did not offer protective immunity following exposure to a lethal dose of SFTSV, and Gn immunization only provided partial protection. The antigen-specific T cell responses to these surface glycoprotein antigens were minimally induced when administered with alum hydroxide adjuvant. Recent research by Kim et al. has introduced an innovative approach using a 24-mer self-assembling ferritin (FT) nanoparticle to present the Gn head region (GnH), significantly boosting immunogenicity (Kim et al., 2023). Mice vaccinated with GnH-FT nanoparticles demonstrated strong neutralizing activities and T cell immunity against Gn, and importantly, aged ferrets immunized with these nanoparticles were fully protected against a lethal challenge with SFTSV (Kim et al., 2023). This suggests that to achieve complete protection, vaccination strategies utilizing SFTSV glycoproteins must invoke strong immunogenicity and stabilize conformational epitopes through multivalent protein complexes that mimic viral particles (Kanekiyo et al., 2013, 2015; Kim et al., 2023). Furthermore, the protective efficacy of neutralizing antibodies against the surface glycoproteins was corroborated by the transfer of immune sera generated through DNA vaccination in a previous study (Kwak et al., 2019). Nonetheless, it is intriguing that while Gc vaccination significantly boosted neutralizing activity against SFTSV, it did not confer protection in the immunized mice in both our current and previous research (Kang et al., 2020). The specific functionality and *in vivo* role of the neutralizing antibodies produced through Gc immunization warrant further investigation.

Our findings also demonstrate that immunization with the NP antigen elicited stronger antibody responses than those triggered by either Gn or Gc immunization. However, these antibodies did not possess neutralizing capabilities. Instead, NP vaccination induced more effective T cell responses and offered superior protection against a lethal SFTSV challenge compared to vaccinations with the Gn or Gc subunits alone (Figures 1, 2). Previous research has confirmed the induction of robust T cell responses and non-neutralizing antibodies following vaccination with DNA encoding the NP antigen (Kwak et al., 2019; Kang et al., 2020), yet such immunization only conferred partial protection against a lethal SFTSV infection (Kwak et al., 2019). Interestingly, our study observed a more pronounced induction of NP-specific antibodies and T cell responses when the subunit antigen was co-administered with the Gc protein (Figure 3). Furthermore, the combination of NP + Gc vaccination yielded the highest protective efficacy among all tested experimental groups in our study. These findings suggest that NP-specific T cell responses, possibly in conjunction with specific antibodies, could significantly contribute to protection against SFTSV infection. Although NP antibodies did not exhibit neutralizing activity, their potential role in antiviral immunity remains to be clarified. Nevertheless, incorporating the NP antigen into vaccine formulations, especially when combined with glycoproteins, may enhance the vaccine’s protective efficacy.

The levels and duration of specific antibodies, including their neutralizing capability, along with T cell immunity, are vital for evaluating vaccine effectiveness and enduring protection against various viral infections, such as SFTSV. Previous research has linked poor serological responses to SFTSV with increased mortality, indicating that a combined impairment of B-cell and T-cell functions disrupts antiviral immunity (Song et al., 2018). This disruption is marked by a lack of specific IgG against the viral nucleocapsid and glycoproteins, attributed to B-cell class-switching failures, underscoring the critical role of SFTSV-specific antibody production in conferring potential protection against the virus. Moreover, longitudinal studies on SFTS patients have shown that SFTSV-specific antibodies can persist for up to 9 years, peaking in neutralizing activity 2 months post-symptom onset (Li et al., 2023). However, these neutralizing antibody levels tend to decrease significantly from 6 months to 4 years post-infection, hinting at a possible reinfection risk within 4 years after the initial SFTSV infection and highlighting the need for additional vaccination. In our study, the peak levels of IgG antibodies and their neutralizing effects induced by vaccination with SFTSV subunit antigens were observed 2–3 months following the final vaccine dose, with a subsequent decline over time (Figure 4). As observed in earlier stage, NP-specific antibodies were generally higher than those against Gn or Gc; however, the dynamics of all antibodies were similar and the neutralizing activity was getting insignificant from 6–12 months post-vaccination. Additionally, we observed a significant decrease in NP-specific CD8^+^ T cell responses 6 months post-vaccination (Supplementary Figure S3). Consequently, all mouse groups vaccinated with the subunit antigens eventually succumbed to SFTSV infection 1 year post-final immunization, suggesting that an optimized SFTS vaccine formulation combining protective antigens with an adjuvant system to ensure long-term humoral and cellular immunity is essential for future vaccine development.

A limitation of our study is the use of IFNAR-KO mice, which lack crucial type I interferon responses to viral infection, potentially affecting the evaluation of specific adaptive immunity, including antibody and T cell responses upon vaccination and/or viral challenge. Utilizing alternative animal models, such as older ferrets or wild type mice temporarily treated with anti-IFNAR antibodies (Reece et al., 2018; Sun et al., 2021), could enrich our findings. Despite this, we observed comparable antibody and T cell responses between IFNAR-KO and wild type C57BL/6 mice (Figures 1, 4), providing valuable insights into the immune response dynamics following vaccination with SFTSV subunit antigens. Secondly, our study utilized structural antigens derived from SFTSV genotype D (KASJH strain), while the challenge virus, 2015-JJ01, belongs to genotype B. SFTSV, endemic in Eastern Asia, displays significant genotype heterogeneity, with at least 6 to 7 genotypes identified (Zu et al., 2022). Considering this diversity, the severity of disease and the efficacy of protection could vary based on the vaccine antigens and the challenge virus derived from different strains (Yu et al., 2019; Yun et al., 2020; Dai et al., 2022; Jeon et al., 2023). Therefore, evaluating the potential cross-reactivity and heterologous protection against diverse genotypes will be crucial in future studies.

## Data availability statement

The original contributions presented in the study are included in the article/Supplementary material, further inquiries can be directed to the corresponding authors.

## Ethics statement

The animal study was approved by Institutional Animal Care and Use Committee. The study was conducted in accordance with the local legislation and institutional requirements.

## Author contributions

SK: Data curation, Formal analysis, Investigation, Software, Validation, Visualization, Writing – original draft, Writing – review & editing. KJ: Data curation, Investigation, Software, Visualization, Writing – original draft. HC: Methodology, Resources, Writing – original draft, Formal analysis. D-EJ: Methodology, Validation, Writing – original draft, Resources. J-GK: Conceptualization, Project administration, Supervision, Validation, Writing – original draft, Writing – review & editing, Funding acquisition, Methodology. N-HC: Conceptualization, Project administration, Resources, Supervision, Validation, Writing – original draft, Writing – review & editing.
