# Supplementary material for: Comparative analysis of the efficacy of vaccines using structural protein subunits of the severe fever with thrombocytopenia syndrome virus
Source: Front Microbiol. 2024 Mar 19;15:1348276. doi: 10.3389/fmicb.2024.1348276 (PMC10985320; doi:10.3389/fmicb.2024.1348276)

Supplementary Table 1

**NP (NCBI Genbank accession no. KP663733.1):**<br>SEWSRIAVEFGEQQLNLSELEDFARELAYEGLDPALIIKKLKETG<br>GDDWVKDTKFIIVFALTRGNKIVKASGKMSNSGSKRLMALQEK<br>YGLVERAETRLSITPVRVAQSLPTWTCAAAAALKEYLPVGPAV<br>MNLKVENYPPEMMCMAFGSLIPTAGVSEATTKTLMEAYSLWQ<br>DAFTKTINVKMRGASKTEVYNSFRDPLHAAVNSVFFPNDVRVK<br>WLKAKGILGPDGVPSRAAEVAAAAYRNL
**Gn (NCBI Genbank accession no. KP663732.1):**<br>DSGPIICAGPIHSNKSADIPHLLGYSEKICQIDRLIHVSSWLRNH<br>SQFQGYVGQRGGRSQVSYYPAENSYSRWSGLLSPCDADWL<br>GMLVVKKAKGSDMIVPGPSYKGKVFFERPTFDGYVGWGCGS<br>GKSRTESGELCSSDSGTSSGLLPSDRVLWIGDVACQPMTPIPE<br>ETFLELKSFSQSEFPDICKIDGIVFNQCEGESLPQPFDVAWMD<br>VGHSHKIIMREHKTKWVQESSSKDFVCYKEGTGPCSESEEKT<br>CKTSGSCRGDMQFCKVAGCEHGEEASEAKCRCSLVHKPGEV<br>VVSYGGMRVRPKCYGFSRMMATLEVNQPEQRIGQCTGCHLE<br>CINGGVRLITLTSELKSATVCASHFCSSATSGKKSTEIQFHSGSL<br>VGKTAIHVKGALVDGTEFTFEGSCMFPDGCDAVDCTFCREFLK<br>NPQCYPAKK
**Gc (NCBI Genbank accession no. KP663732.1):**<br>CDEMVHADSKLVSCRQGSGNMKECVTTGRALLPAVNPGQEA<br>CLHFTAPGSPDSKCLKIKVKRINLKCKKSSSYFVPDARSRCTSV<br>RRCRWAGDCQSGCPPHFTSNSFSDDWAGKMDRAGLGFSGC<br>SDGCGGAACGCFNAAPSCIFWRKWVENPHGIIWKVSPCAAW<br>VPSAVIELTMPSGEVRTFHPMSGIPTQVFKGVSVTYLGSDMEV<br>SGLTDLCEIEELKSKKLALAPCNQAGMGVVGKVGEIQCSSEES<br>ARTIKKDGCIWNADLVGIELRVDDAVCYSKITSVEAVANYSAIPT<br>TIGGLRFERSHDSQGKISGSPLDITAIRGSFSVNYRGLRLSLSEI<br>TATCTGEVTNVSGCYSCMTGAKVSIKLHSSKNSTAHVRCKGDE<br>TAFSVLEGVHSYTVSLSFDHAVVDEQCQLNCGGHESQVTLKG<br>NLIFLDVPKFVDGSYMQTYHSTVPTGANIPSPTDWLNALFGNG<br>LSR

Supplementary Figure 1

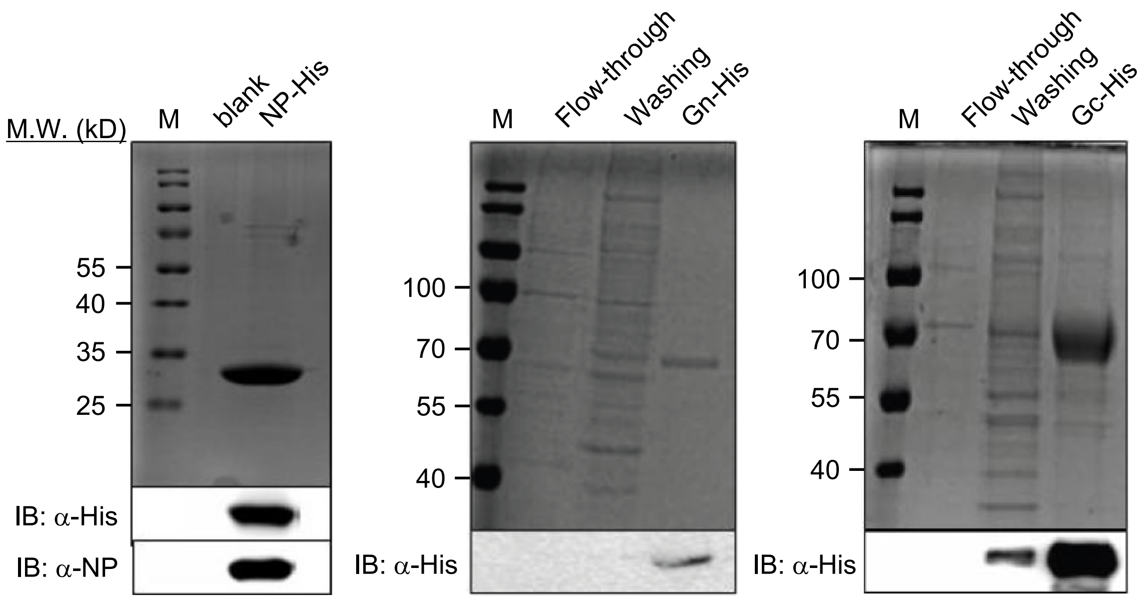

Supplementary Figure 2

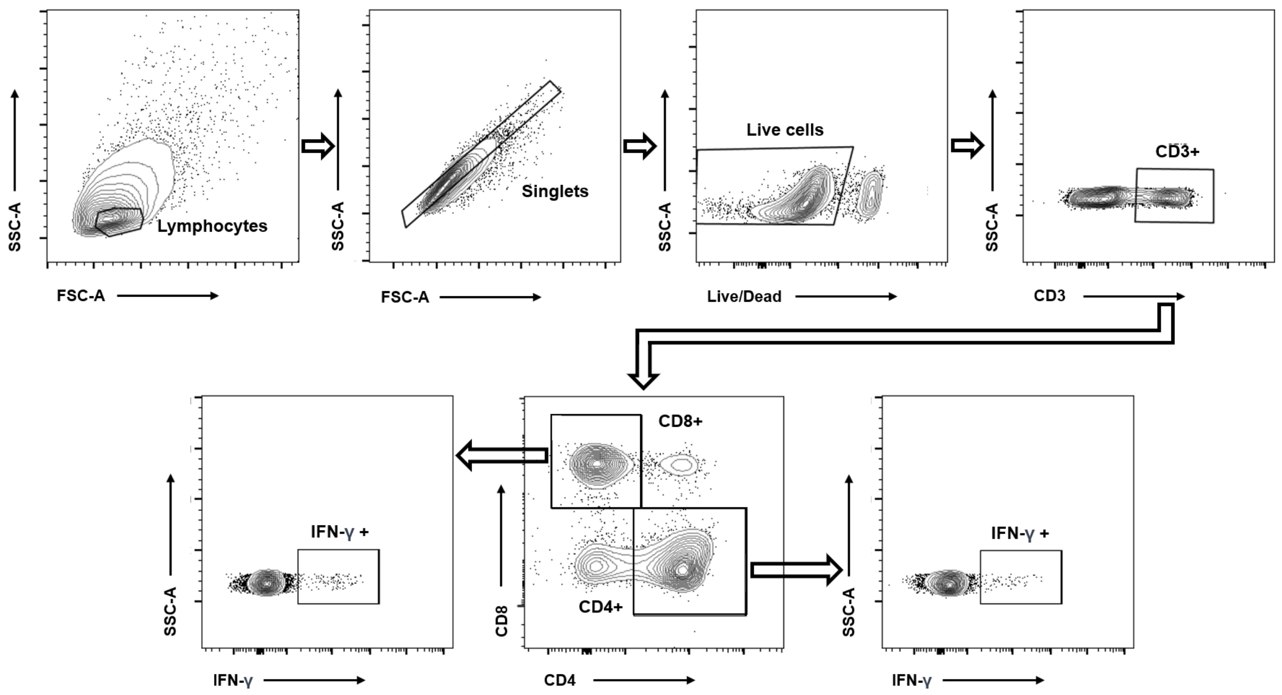

Supplementary Figure 3

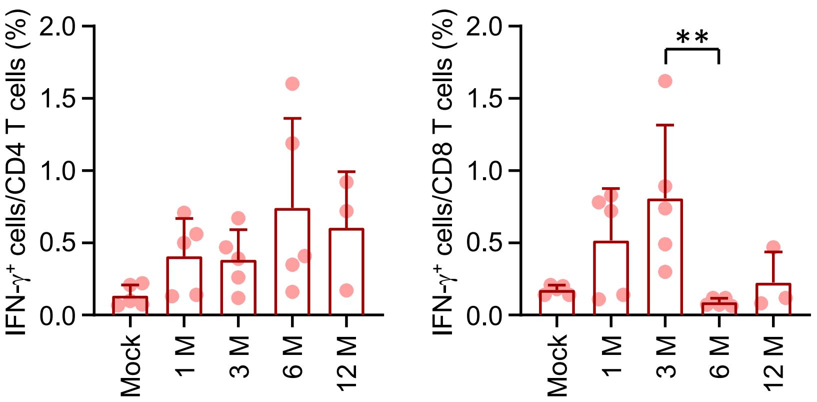

Supplement: Supplementary file 1 [file Data_Sheet_1.PDF]
